# A Change Management Approach to Promoting and Endorsing Ergonomics within a Dental Setting

**DOI:** 10.3390/ijerph192013193

**Published:** 2022-10-13

**Authors:** Rami J. Anshasi, Adi Alsyouf, Fahad Nasser Alhazmi, Abeer Taha AbuZaitoun

**Affiliations:** 1 Prosthodontics Department, Faculty of Dentistry, Jordan University of Science and Technology, Irbid 22110, Jordan; 2 Department of Managing Health Services and Hospitals, Faculty of Business Rabigh, College of Business (COB), King Abdulaziz University, Jeddah 21991, Saudi Arabia; 3 Department of Health Services and Hospital Administration, Faculty of Economics and Administration, King Abdulaziz University, Jeddah 21589, Saudi Arabia; 4 College of Dentistry, Jordan University of Science and Technology, Irbid 22110, Jordan

**Keywords:** ergonomics, dentistry, musculoskeletal disorders (MSDs), static posture, risk factors, Kotter change model

## Abstract

Musculoskeletal pain and disorders (MSDs) constitute a well-recognised occupational hazard to the dental community. Fortunately, they are preventable. Dental office ergonomics plays a key role in addressing these musculoskeletal challenges. An ergonomic improvement project based on Kotter’s eight-step change model management theory was implemented within a dental practice. The project provided valuable evidence-based ergonomic interventions to prevent or reduce MSDs. The task force motivated staff to engage in strategies and interventions to enact an ergonomic vision. This case study represents an action plan to guide this ergonomic change. The key results of this project were an evidence-based ergonomics health promotional brochure, reduced sick leave attributable to MSDs, and workplace ergonomic checkpoints. The ergonomic change model represents an ongoing process where innovative trends and evaluative methods can be supported. Research limitations and practical implications were acknowledged.

## 1. Introduction

Work-related musculoskeletal diseases (MSDs) are considered inherent occupational hazards in modern dentistry. They are a problematic health issue among dental professionals [1]. Some studies have revealed a high incidence of MSDs among dental personnel (72.6%) [2]. The Center for Disease Control and Prevention describes musculoskeletal disorders as injuries or disorders of the muscles, nerves, tendons, joints, cartilage, or localised blood circulation in upper and lower limbs, neck, shoulder, and back [3]. Examples of MSDs include carpal tunnel syndrome, de Quervain’s disease, Guyon’s syndrome, Raynaud’s syndrome, cubital tunnel syndrome, bursitis, thoracic outlet syndrome, myofascial pain disorder, cervical spondylosis, back disc problems, sciatica, tension neck syndrome, trapezius myalgia, rotator cuff impingement, and trigger finger [4]. The most afflicted region is the neck, followed by the shoulder, the lower back, and the upper back [5].

Dental staff spend their workdays in stiff, inflexible positions, carrying out procedures with high precision and focus in a very narrow, enclosed space, the patient’s mouth. Because the profession requires steady hands, strained stances, and static work, occasional pain in staff’s neck, shoulder, and back areas is expected. Those rather seemingly not harmful working positions are repeated so often that they eventually may result in injuries [6].

Occupational risk factors that contribute to MSDs include deviated working postures, repetitive motion, forceful exertions, exposure to vibrating tools, prolonged work duration, mechanical stress, poorly designed dental equipment, and other factors [7]. MSDs are considered a multifactorial problem, which explains why the effect of a single ergonomic intervention is limited [8].

MSD symptoms include difficulty performing routine movements, motion limitation, tingling numbness, twitchy movements, weak grip, fatigue, pain, cramps, stiffness, and tenderness of affected body parts [9]. MSDs can be mild and infrequent but can be severe, chronic, and debilitating [10].

Established MSDs lead to difficulty in task performance, absenteeism, reduced productivity, decreased job satisfaction, financial losses from lower working hours, medical expenditure for therapy, and early retirement by dental professionals [8].

Several ergonomic interventions were found to prevent MSDs among dental professionals [11]. Ergonomics studies people at work and how their workplace environment is modified to fit the worker’s capabilities. It is a way to work smarter and not harder by suiting the job to the worker and not the opposite [12]. Dental ergonomics, or ergo-dentistry, is concerned with designing dental products and procedures for optimum dental personnel’s well-being and overall system performance [13]. It puts dental professionals first, ensuring compatibility between them and their workplace. It is incumbent for dental organisations, colleges, hospitals, centres, and clinics to adopt an “ergonomics mindset” and take an active role in this initiative. Essential ergonomics education is seldom embraced in dental schools [14]. However, it would be better for future dental graduates to include dental ergonomics in the academic curriculum [15].

This project aims to directly inform dental personnel within the workplace and enable them to evaluate their practices regarding ergonomics. This knowledge transfer helped staff identify occupational MSD hazards and how to make workplace changes based on evidence-based ergonomics health promotion recommendations. It highlights how the change management theory of John Kotter, expressed in his 8-step model, could help dental personnel make the transition to better and smarter ways of working ergonomically and ultimately reducing the risks associated with MSDs. Staff were encouraged to engage in the project that aimed to reduce and prevent MSDs and build up a maintainable, ergonomic dental culture. Dental ergonomics literature is a wide topic area and has been the subject of numerous documented studies worldwide [1,4,8,11,16,17]. The Kotter model has been used as an implementation guide to lead change efforts in various educational and healthcare settings [18,19,20,21,22]. This action research paper contributes to the literature by choosing the Kotter model as a framework to empower individuals and organisations to implement an ergonomic initiative to tackle MSDs.

## 2. Materials and Methods

### 2.1. Embracing Ergonomic Interventions

A 30-chair dental facility that provides services to the community is currently undergoing organisational change to implement an ergonomics intervention program. The dental centre employs 45 dental personnel, many of whom experience MSDs. Implications were not just limited to regular sick leave, but also included reduced productivity, and decreased working hours. Staff members who had MSDs needed various treatment modalities, ranging from over-the-counter pain medications, physiotherapy sessions, and splinting to even surgery, such as carpal tunnel surgery. The staff members acknowledge that inadequate control of MSDs and the means of their prevention should be addressed. Effective measures to avoid MSDs should be accepted as a norm and a routine standard by dental health workers.

This action plan project applies Kotter’s eight-step change model to implement ergonomic prevention strategies to develop a workplace culture free of MSDs. The project’s dual goals are to reduce and prevent MSDs and to enhance a dental ergonomic mindset. John Kotter’s change management model was adopted because it is a simple progressive model that emphasises adapting to change, and the transition between its stages is easier [18]. It is a highly adaptable framework for a health care setting [19]. Key ergonomic interventions and their relevant effects will be based on the most current state of literature review research on ergonomic interventions [8]. Each phase in Kotter’s change model will be linked with examples from ergonomic interventions, considering the limitations and shortcomings. The ultimate purpose is to gradually bring the best ergonomics improvement ideas into the dental practice to reduce MSDs by applying the Kotter change model.

Kotter studied the change efforts of more than 100 organisations before formulating his model for successful change. He proposed eight stages to produce a successful change in organisations [20]. The eight steps are grouped into three distinct phases (Table 1). The process focuses on the “see-feel-change” approach by relating to peoples’ emotions to successfully change their behaviours [21].

There was a six-month interval allocated for this project. A Gantt chart was helpful to keep track of the project timeline and the progress intended.

We thoroughly reviewed dental ergonomic interventions that aim to reduce or prevent MSDs. A research action plan design was created to implement such interventions in a dental setting, using the phases in the Kotter model. Action research is a reflective process of ongoing problem solving by a researcher who collaborates with others in a team to improve how they address issues in their organisation [22]. Carrying out a research action project provides a methodology with effective cycles of planning, acting, evaluating, and reflecting, ultimately generating knowledge and experience that will shape the future direction of further research [23]. Action research uses knowledge from problem-solving in reality [24,25]. This research article integrated a qualitative case study methodology to depict the ergonomic change process that took place. An interventional case study design can help show how a planned organisational initiative advances [26]. Our hypothetical assumption is that adopting Kotter’s change management model will be constructive in the ergonomic change effort. The ergonomic initiative applied Kotter’s 8 steps, which are as follows: establish a sense of urgency, form a powerful guiding coalition, create a strategic vision, communicate the vision, empower others to act on the vision, create short-term wins, consolidate improvements, and institutionalise new approaches.

This study took place in a 30-chair dental facility, with 45 dental and allied dental staff representing our study’s sample. Two of the authors were part of the five -member ergonomic team that led this initiative and selected various tools and strategies to improve the status quo dental ergonomics. Their duties ranged from allocating educational resources, creating brochures, arranging training sessions, applying fishbone analysis, tracking the 6-month project by a Gantt chart, and evaluating the ergonomic change. The following two methods were used to appraise the outcomes or the impact of Kotter’s change model:

(i) A 12-item checklist was developed in the project’s first phase, based on evidence-based ergonomic interventions mentioned in the literature review [11]. The preliminary data from the checklists will serve as baseline information for capturing the ergonomic compliance witnessed. The participants responded to each item on the checklist with simple “yes/no” responses. The checklist is shown in Table 2.
(ii) Comparing the number of sickness absence rates due to MSDs before and after implementing the ergonomic change was another evaluation method. Sick leave data were obtained from electronic medical records, and a prospective drop in sick leave rates due to MSDs was considered an ergonomic improvement [16].

#### 2.1.1. Step 1. Create a Sense of Urgency

A creator of urgency for the ergonomic change could be affirming the numbers of staff with MSDs in the institution. This will prompt the dental staff to start open discussions about MSDs and how to take actions and consider techniques to work ergonomically. The staff’s deposition or testimonial about the course and impact of a musculoskeletal problem and sharing their personal stories is a powerful option to create such a sense of importance. MSDs result in reduced work performance, reduction in routine activities, pain that prevents staff from going to work, and early retirement. Taking analgesics, physiotherapy, and even surgical interventions may be needed to overcome this affliction. Each dental health worker must understand the physical demands of the practice.

A more interactive approach would be arranging a visit by an occupational therapist or a dental ergonomics expert to the dental institution. A convincing dialogue with management about empirical ways to reduce and prevent MSDs would result in financial benefits, increased productivity, and better quality of work. High staff turnover because of MSDs is detrimental because skilled dental personnel may be difficult to replace. A combination of staff’s MSD complaints, MSD-related sick leave, managerial involvement, open communication among colleagues, and an ergonomist’s invitation creates a catalyst for urgency.

#### 2.1.2. Step 2. Build an Ergonomic Coalition

An essential step to promote ergonomics in the dental organisation is to obtain management commitment and sponsorshiManagement must participate in goal setting and resource allocation in any ergonomic process and be a part of the ergonomic team. The guiding coalition or team comprises key staff representatives interested in deploying ergonomics. The five-member team included a consultant dentist, a clinician, a dental assistant, a hygienist, and a dental procurement manager. Team members represented a good mix from different levels and possessed distinct skill attributes. Among these attributes were familiarity with ergonomic knowledge, leadership roles, enough stature, credibility and official authority [27,28]. Other qualities needed included persuasiveness to talk the staff into protecting themselves against MSDs, confidence in finding solutions, thoroughness in probing staff’s doubts, and imaginative communication using various channels [29,30]. Perhaps more important for the ergonomic task force is to have an “analysis-think-change” mindset by leading, encouraging and motivating colleagues to achieve the vision of sound ergonomic practices [21]. The team played a key role in driving ergonomic plans, communicating them to staff and enabling or gradually procuring ergonomic resources.

#### 2.1.3. Step 3. Form the Ergonomic Vision

The vision is to build an ergonomics-sensitive culture within the dental workplace to prevent MSDs. Kotter stated that it is likely to be too inaccessible if the vision cannot be communicated in five minutes or less. A good vision statement can be made clear in a “sixty-second elevator ride” [31]. Any desired ergonomic change should entail a future vision for a working environment with avoidable MSDs. The project should begin with a clear vision of the presumed change and proceed with actions to make this change happen [32]. Meetings, ergonomic educational sessions, and conversations with staff are key moments that can be used to highlight a sense of urgency, encourage staff to buy in, build a guiding team and create better practical ergonomics [20]. These three steps help initiate an ergonomic climate.

#### 2.1.4. Step 4. Communicate the Vision and the Initiatives

**Figure 1:**
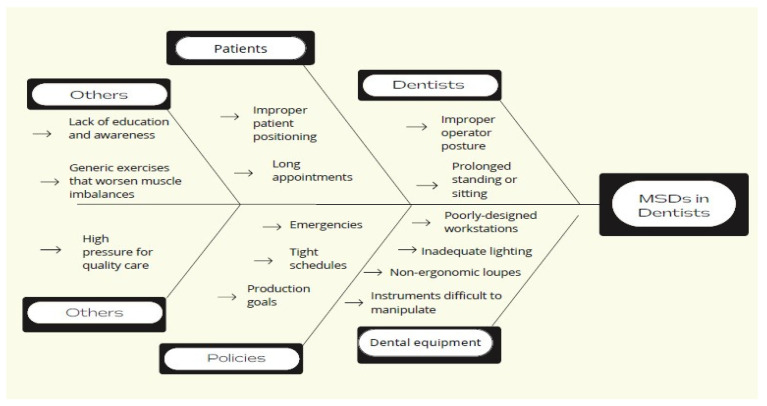
Fishbone diagram.

Actions and strategic initiatives need to be taken to achieve the ergonomic vision [20]. The task force formulated the following plan that must be executed:

1. Develop a user-friendly information brochure entitled “MSDs Prevention among Dental Personnel”. (See Appendix A).
2. Identify available educational resources and construct training materials, such as videos, posters, and lectures.
3. Take advantage of periodic staff meetings and education sessions, such as the dental journal club, to identify ergonomic behaviours.
4. Design a fishbone diagram to help dental personnel understand the causes of musculoskeletal problems. (See Figure 1).
5. Review various strategies or recommendations advocated to reduce ergonomic problems.
6. Conduct research on ergonomic dental equipment to assist in the funding decisions required. Managers have a crucial role in supporting and enabling the adoption of ergonomic technologies.
7. Look to others who have led similar ergonomic projects. For instance, the team reviewed a comparable ergonomic program proposed by the Occupational Safety and Health Administration (OSHA). The program included the following six elements: management leadership, hazard information and reporting, job hazard analysis and control, ergonomic awareness training, MSD management, and program evaluation [24].
8. Encourage and involve others to suggest and discuss ergonomic improvement ideas.
9. Create a Gantt chart to monitor project progress and document ergonomic progress and obstacles.

These coordinated initiatives can turn the ergonomic vision into a reality.

#### 2.1.5. Step 5. Empower Staff to Act on the Vision and Remove Obstacles

Staff were empowered to adapt ergonomic interventions. The ergonomic team that endorsed the project provided knowledge concerning how these interventions mitigate MSDs, what staff training is needed, and the ergonomic expenses. The literature review presented different ergonomic interventions. Ergonomic dental chairs, magnification loupes, prismatic spectacles, dental instruments, and educational training sessions in ergonomics had a significant positive impact on MSDs [8]. Ergonomic dental chairs, such as the saddle stool and dental chairs with arm support, provided comfort, maintained the natural curvature of the lower back, and improved the working posture [33,34]. The use of magnification loupes improved the working posture and significantly reduced symptoms of MSDs [35,36]. Prismatic spectacles significantly and positively changed the working posture, reduced MSD complaints, and simplified daily dental work [37,38]. Proper selection of lightweight dental instruments with a wide diameter, rather than heavyweight instruments with a narrow diameter, significantly lowered MSD symptoms [39]. Hard-wearing automated hand tools should be incorporated instead of manual ones [40]. Theoretical and practical educational training courses in ergonomics included in-person coaching, circulating pamphlets, evaluating workstation conditions, modifying working postures, taking periodic micro-breaks, and explaining chair side stretching movements and strengthening exercises by a physiotherapist or an occupational therapist. Research-based knowledge gained through such training programs reduced the prevalence of MSDs and improved workplace ergonomics [41,42,43]. Other considerations to “ergonomize” the dental office include practising four-handed dentistry, appropriate lighting, indirect mirror viewing, good temperature and ventilation, alternating between sitting and standing, scheduling appointments to interchange between easy and difficult cases, selecting patient chairs with cervical rests, cordless handpieces, and voice-activated charting [40,44,45,46,47,48,49,50]. Acquiring advances such as intuitive extraction kits, spinning self-cleaning dental mirrors, dry field illuminators, personal LED headlights, laser, Cad-Cam technology, ergonomic gloves, and other innovations indicate the wide scope and continuous evolution of dental ergonomics [51,52,53,54]. New ergonomic skills will change custom practices, and staff need to familiarise themselves with such skills in the workplace.

#### 2.1.6. Step 6. Create Quick Ergonomic Wins

Kotter suggested that short-term wins energised the project and reinforced momentum for success [55]. Successfully deploying early and easy ergonomic improvements is important before choosing to implement costly dental delivery systems. For example, short-term wins may include attending ergonomic training sessions, choosing alternate instrument grips, improving body posture, chair side stretching, sequencing treatment schedules, wearing loupes, and reducing MSDs. Achieving short-term wins enlightens the sceptics, engages staff, reveals benefits, and helps evaluate the progress of the process [32]. Any evidence that demonstrates ergonomic progress must be made clear and visible to all staff to build on the correct ergonomic practices and improve them. Such positive ergonomic results were delivered to staff by the guiding team in official meetings, coaching sessions, and casual conversations.

#### 2.1.7. Step 7. Build on the Ergonomic Change

Organisational change could be a slow process and may take considerable time to maintain [21]. Slowly implementing ergonomic changes into the practice is necessary, and these short-term wins are only the beginning of what needs to be achieved in the long term [31,32]. Any change process represents a continuous improvement cycle. Similarly, ergonomic change in the dental setting has no ultimate end. There are always new ergonomic approaches to consider and seek.

#### 2.1.8. Step 8. Anchor the Ergonomic Culture 

When operating ergonomically becomes the norm within the core of the dental setting, it could be said that the change was implemented. Consolidating the ergonomic change requires recognising and rewarding the behaviours of all staff involved in the process. Harvesting the fruits of working comfortably, experiencing better productivity, and preventing and reducing MSDs will help sustain the change in practice.

## 3. Project Outcomes and Discussion

This section provides a concise and precise description of the experimental results, their interpretation, and the experimental conclusions that can be drawn.

The project was evaluated using two assessment tools. Data were collected using checklists to measure ergonomic improvements in dental office practice status. The ergonomic team collected the data. The checklist acts as an initial assessment of ergonomic improvement post-implementation at the workplace after in-service education of MSD hazards, and ergonomic awareness has been carried out. An important opportunity to invest in is exercising caution in purchasing ergonomic dental equipment, and such a measurement is tangible and specific. Another evaluation tool used in the project was reducing MSD complaints and their sick leave or absence levels. Other suggestive screening tools utilised in ergonomic programs may include walk-through observational surveys of dental facilities, interviews with dental professionals, and assessments by surveys. However, these measurements were not evaluated within the limits of the time framework of the project.

After six months, the findings of the ergonomic team using checklists and sick leave as tools were analysed. Forty-five dental personnel participated in the ergonomic intervention program. The majority were dentists (n = 30, 67%), and fifteen were dental assistants and hygienists (n = 15, 33%). Post-intervention sick leave rates because of MSDs fell from 7% to 5% during the 6-month interval compared to last year. Information on sick leave or absence hours taken by staff attributed to MSDs was extracted from electronic medical records. This crude indicator shows the ramification of ergonomic measures and staff engagement. Some studies propose that dental professionals resort to 1–7 instances of sick leave per year caused by MSDs [56]. Absenteeism due to MSDs does not only negatively affect the life aspects of professionals but also takes a toll on their organisations. Reduced productivity, cancellation of appointments, rescheduling, colleague overload, staff turnover, and other economic costs are MSD burdens to dental employers [57,58,59]. Hence, Kotter’s steps address organisational ergonomics (workflow, appointment scheduling, and policies), as well as the ergonomics of dental professionals [60].

**Figure 2:**
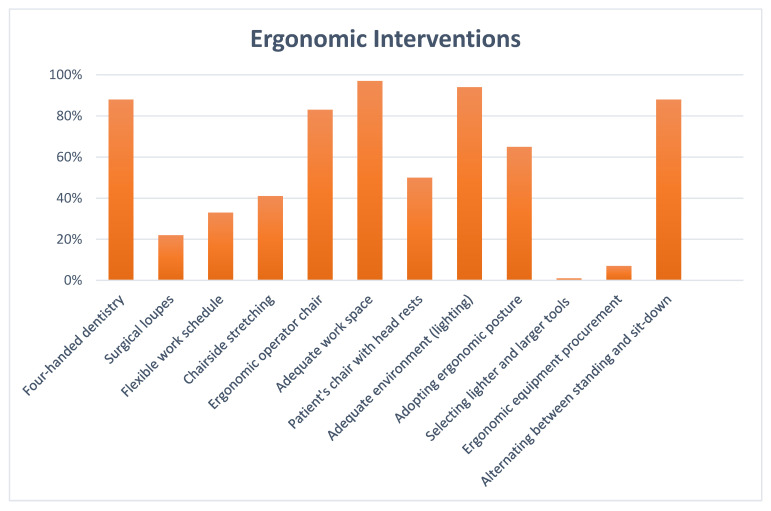
Bar chart showing dental staff checklist results.

When collecting data using checklists, the guiding team received positive comments from staff who expressed good awareness and interest in incorporating sound ergonomic principles into their daily practice. Conducting these ergonomic assessment checkpoints helps reveal sources of ergonomic risks and identify areas for improvement. The checklist results are shown in Table 3 and illustrated by a bar chart in Figure 2.

Eighteen dental staff members (41%) performed chair-side stretching, an intervention that can be easily carried out. Periodic stretching stimulates blood circulation, increases the production of synovial fluid in joints to keep them moving smoothly, releases aching trigger points, and helps muscles to achieve a productive range of motion [61].

The results of the checkpoints also show that less ergonomic sophistication was apparent, given that there was low employment of surgical loupes and less notable newly purchased ergonomic equipment. Only ten dental personnel (22%) used magnification loupes. Staff may resist using magnification loupes for several reasons. The considerable price of loupes and their longer learning curve may partly explain this finding [62]. It is important to use dental loupes as they improve posture, increase visual acuity and reduce MSDs [63,64]. The financial barrier against using loupes could be removed if management assists staff with financing by deducting some of their employees’ wages over a year to cover the costs of loupes, paying for a share of the loupes, or agreeing that the loupes could be claimed by the employee after one or two years [65].

Within the project’s timeline, seven sorts of newly purchased dental products had noteworthy ergonomic features. It makes sense as management carries thorough research before investing in new ergonomic equipment [66]. These products included double-sided, magnified mirrors, padded periodontal instruments with thick silicone handles, cordless prophylaxis handpieces, high-speed handpieces with fibre-optic options, self-aspirating dental cartridge syringes for analgesia, ergonomic gloves, and a recently developed rubber dam system.

Double-sided mirrors achieve tissue retraction and indirect vision together [67]. Using instruments with a thick diameter (12–14 mm) silicon handles causes lower musculoskeletal strain [68]. Cordless prophy handpieces with a thick diameter, a tactile grip and light weight with essentially no cord pullback helped reduce workload and fatigue [48]. Fibre-optic equipped handpieces optimise light on the dental site, resulting in less strain for dental staff [69]. Ergonomic self-aspirating syringes are efficient compared to poorly controlled, conventional syringes during aspiration [70]. Light pliant gloves that fit snugly and do not apply pressure on the hands provide comfort [44,71]. Modern rubber dam isolation systems exhibit quicker handling time and more effective isolation than conventional systems [72].

Thirty dental members (67%) claim to work on a tight schedule. Tight work schedules have been risk factors for MSDs [73]. One justification behind the tight schedules seems to be that those booking appointments may not consider how many patients the dental professional can treat in one day, leading to workload [74]. Alternating between easy and tough cases, as well as short and lengthy sessions, giving the dental professionals more control over their working circumstances, varying the sequence of procedures, taking micro-breaks, and lessening patients’ follow-up time or the daily number of patients, represent preventive solutions to busy agendas [74,75,76].

The majority of staff, 40 (88%), reported practising four-handed dentistry whenever applicable. Four-handed dentistry helps deliver dental care ergonomically and efficiently [77]. Four-handed dentistry is a teamwork approach, where dental staff work together in an ergonomically planned manner to improve efficacy, quality of care, and comfort [78] This proper layout promotes ergonomics and focuses on efficiently swapping tools between the operator and the dental assistant [79].

Concerns about adopting ergonomic posture in clinics were still perceptible and mainly attributed to efforts to change old working habits and work slowly. Thirty staff members (66%) reported attaining an ergonomic posture during work. The ergonomic or balanced, recommended posture features the oral health professional sitting as close as possible to the patient with their head inclined slightly forward, an upright torso, horizontal shoulders (not raised), arms close to the body, and both feet supported flat on the floor, and with thighs parallel to the ground, forming 90° with the legs [80,81,82].

The ergonomic project revealed clinics that had inadequate workspace or poor lighting. These clearly represented ergonomic strain. Staff complained of one clinic with a cramped workspace. Inadequate workspace will cause needless movement of staff, delay in completion of appointments, and an unfavourable psychological effect on staff [83]. Two other clinics presented with poor lighting. Poor lighting exposes dental staff to eye fatigue, headache, weariness, and psychological stress [84]. Both of these situations were resolved.

Ten out of sixty operator stools warranted repair or replacement. Ergonomic dental stools should be adjustable and provide lumbar support and armrests [85]. Specialised saddle stools are also considered ergonomic, offering a convenient natural lower back curve and a correct shoulder neck posture [33].

The percentage of staff that alternated between sitting and standing posture was 88% (n = 40). It was found that combining the sitting and standing working postures reduces fatigue and discomfort [86]. Standing minimises pressure on the back, and sitting transfers weight to the seat; hence, alternating between postures allows muscle workload to move from one body area to another [87].

Fifteen out of thirty patients’ dental chairs had no adjustable headrests. Adjustable headrests support effortless access for the dental operator, and multi-articulated headrests improve viewing by positioning patients’ heads along three axes [88].

Newly purchased dental tools that had lighter weights and larger diameters within the project’s timeline were periodontal scalers and curettes. The research supports the belief that lighter and wider instruments produce less pinch force and muscle load, i.e., better ergonomics [60,89].

The budget factor for the management may limit the incorporation of more costly ergonomic equipment and expensive hands-on training sessions. However, the dental staff may consider purchasing personal equipment, namely, magnification systems for themselves. Besides the quality, infection control, and journal club teams present in the centre, the ergonomic team laid the basis for further development in the future.

The notion that “in order to be a dentist, there must be an inevitable ergonomic compromise” should change. One interesting perspective from a dental teammate was as follows: “I know I have to practice ergonomically more often to lessen fatigue incidents. But when will this ergonomic practice become habitual?” Practice is the key. In Kotter’s words,” let it stick!”.

## 4. Conclusions

MSDs are frequently reported by dental personnel, given the occupational risk factors. They may involve the lower back, hands, shoulders, or neck. They are cumulative and may build over the years. However, they can be avoided by practices and behaviours that are considered ergonomic. This action plan study sought to employ Kotter’s structured eight-step framework for adopting an ergonomic workplace culture to prevent the likelihood MSDs. Kotter’s model can help clarify and craft the new ergonomic practices by seizing opportunities and addressing challenges. Kotter’s model, expressed in his eight-step process (developing urgency, building a guiding team, creating a vision, encouraging staff to buy-in, enabling action, creating short-term wins, encouraging consistency and permanent behaviours) represented a working framework to guide ergonomic recommendations and practice changes for dental staff. Promising outcomes of the change process were achieved, including the release of an informational brochure, a 2% reduction in MSD-related sickness absence rate, and ergonomic awareness, behaviours, and practice improvements.

### 4.1. Research Limitations/Implications

This project was implemented in a dental workplace within six months. Changes in the ergonomic status of practice were measured using MSD-related absence levels and checklists. These could be considered “short-term” wins, as Kotter implied. However, different evaluation instruments, such as surveys, interviews and observational tools, could be used. Many factors, such as general health, number of patients treated per day, and detailed work positions, were not considered. More time and resources may be needed to analyse these factors. Dental ergonomics has a wide scope indeed. Costly ergonomic equipment or resources and being unable to change old working behaviours or habits could also be limiting factors. However, there is great potential for simpler ergonomics.

### 4.2. Practical Implications

A change management model could be indicative when considering ergonomic practice changes in the dental workplace. Kotter’s change model was chosen for this study. The steps within the model aid us in enabling ergonomic practice change and overcoming any barriers to such a practice. Ergonomic knowledge and interventions are critical to enable change to happen. Future ergonomic plans that may be proposed may include learning web-based portals, online websites, and hands-on training. The ergonomic process is continual because there is always room for improvement with various tools of evaluation.

## Figures and Tables

**Table 1:** John Kotter’s eight-step change model.

Kotter’s 8-Step Change Model	
Step 1: Urgency Step 2: Coalition Step 3: Vision	Creating a climate for change
Step 4: Communicate Step 5: Empower Step 6: Wins	Engaging the organisation
Step 7: Consolidate Step 8: Anchor	Implementing the change

**Table 2:** Ergonomic checklist for dental staff.

Item	Ergonomic Feature	Yes	No
1	Breaks and chair side stretching exercises during the workday		
2	Adequate work environment space		
3	Inadequate environment (lighting, temperature, humidity)		
4	Flexible work schedule		
5	Ergonomic operator chair		
6	Patient chair with adjustable headrests		
7	Using surgical loupes		
8	Practising four-handed dentistry		
9	Changing postures frequently (alternating between standing and sit-down dentistry)		
10	Selecting larger and lighter handles of dental instruments		
11	Considering the purchase of ergonomic dental equipment		
12	Adopting ergonomic posture		

**Table 3:** Dental staff ergonomic checklist results.

Ergonomic Checkpoint Applied	n */(%)	Brief Description of Intervention Effect
1- Chairside stretching	18—(41%)	Reduces MSDs and has a positive effect on symptoms
2- Inadequate workspace	1—(3%)	Narrow workspaces are risk factors for MSDs
3- Inadequate environment (lighting)	2—(6%)	Good lighting increases the operator’s clarity and accessibility
4- Surgical loupes	10—(22%)	Loupes influence the working posture
5- Tight schedule	30—(67%)	Flexible scheduling provides a sufficient recovery time
6- Ergonomic equipment procuring	7—(7%)	Enhances comfort and efficiency
7- Four-handed dentistry	40—(88%)	Promotes efficiency and reduces stress
8- Ergonomic posture	29—(65%)	Proper posture maintains good health
9- Non-ergonomic operator chair	10—(16%)	Improves the working posture
10- Alternate between standing and sitting	40—(88%)	An effective tool to prevent MSD injuries
11- Patients’ chairs with no adjustable headrests	15—(50%)	Maximises patient access
12- Tools with lighter and larger handles	2—(2%)	Affects hand muscle load

* Estimated number of staff, workstations, or tools ergonomically involved.

**Figure A1:**
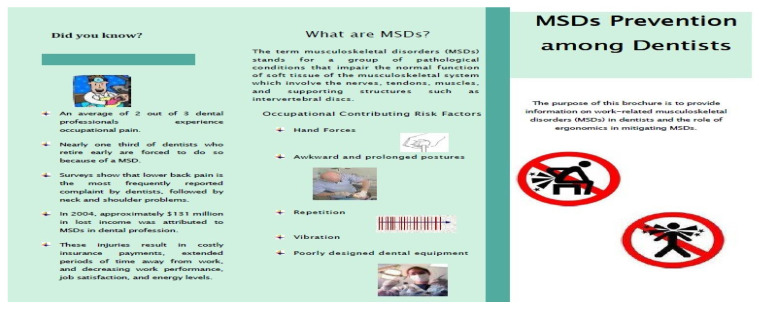

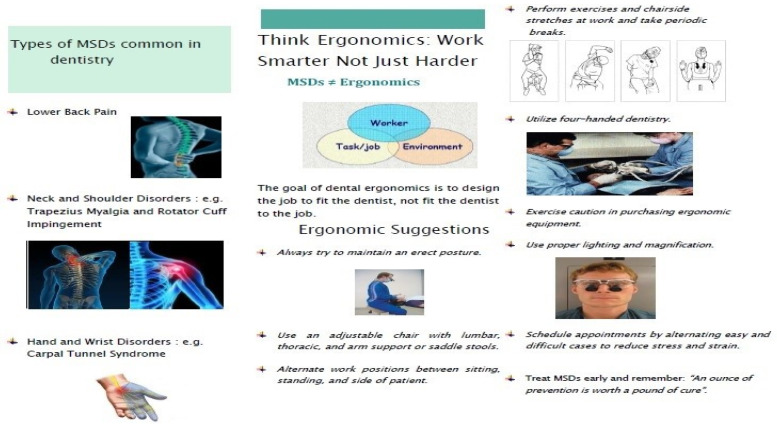
Informational brochure identifying work-related MSDs among dental personnel, their risk factors and evidence-based preventive ergonomic approaches.

## Data Availability

The data presented in this study are available on request from the corresponding author.
